# Prolonged Clinical Benefit with Futibatinib in a Patient with FGFR Inhibitor–Pretreated *FGFR2* Fusion–Positive Intrahepatic Cholangiocarcinoma: Case Report

**DOI:** 10.2147/OTT.S434449

**Published:** 2024-06-13

**Authors:** Paolo D d’Arienzo, Alan R MacDonald, Virjen Patel, Yuk T Ma, Rille Pihlak, Naureen Starling

**Affiliations:** 1 GI Cancers Unit, The Royal Marsden NHS Foundation Trust, London, UK; 2 Clinical Radiology Department, The Royal Marsden NHS Foundation Trust, London, UK; 3 Department of Oncology, University Hospitals Birmingham NHS Foundation Trust, Birmingham, UK; 4 Department of Oncology, St Bartholomew’s Hospital, London, UK; 5 Division of Clinical Studies, Institute of Cancer Research, London, UK

**Keywords:** cholangiocarcinoma, FGFR inhibitor, targeted therapy, futibatinib

## Abstract

Multiple FGFR inhibitors have demonstrated significant activity in pretreated advanced *FGFR2* fusion–positive intrahepatic cholangiocarcinoma. The irreversible pan-FGFR inhibitor futibatinib has the potential to overcome acquired resistance to ATP-competitive FGFR inhibitors in a subset of patients. We present a case of prolonged clinical benefit using FGFR inhibitors sequentially, initially an ATP-competitive inhibitor followed by futibatinib upon progression, for a total of 36 months of FGFR-targeting therapy. This case supports sequential FGFR-targeting therapies for *FGFR2* fusion–positive cholangiocarcinoma, with futibatinib acting as rescue therapy after failure of ATP-competitive inhibitors.

## Introduction

In 7%–15% of intrahepatic cholangiocarcinomas (iCCAs), rearrangements/fusions in the *FGFR2* gene are detectable as an oncogenic driver.^1^ Several oral FGFR inhibitors have been developed, including ATP-competitive inhibitors with activity against different FGFR receptors (pemigatinib, infigratinib, derazantinib, erdafitinib, Debio1347), irreversible pan-FGFR inhibitors (futibatinib, also known as TAS-120, and KIN-3248) and an irreversible FGFR2 specific inhibitor (RLY-4008).^2–4^ Response rates of 20%–50% have been reported in phase I–II clinical trials with patients with advanced cholangiocarcinoma who had progressed on first-line platinum-based chemotherapy.^2^,^5–9^

The FIGHT 202 trial led to FDA and EMA approval for the use of pemigatinib in pretreated advanced cholangiocarcinoma with *FGFR2* fusion/rearrangements. Overall response rate (ORR) was 35.5%, median progression-free survival (mPFS) 6.9 months, and median duration of response (mDOR) 7.5 months.^5^ In the same setting, treatment with derazantinib was associated with an ORR of 21.8%, mPFS of 7.8 months, and mDOR of 6.4 months.^7^ FOENIX-CCA2, a phase II trial of futibatinib in chemotherapy-pretreated *FGFR2*-rearranged iCCA with no previous FGFR-targeting therapy, has recently been reported with ORR 42%, mDOR 9.7 months, and mPFS 9 months.^9^

Unfortunately, the emergence of acquired resistance to ATP-competitive FGFR inhibitors is common, and limits the duration of clinical benefit derived. On-target resistance mechanisms include the kinase domain gatekeeper mutations p. V565F, I, or L and molecular brakes, such as p. E566A, p. N550D, H, K, or T. Molecular brake mutations cause a rise in kinase activation, which interferes with the inhibitory effect of FGFR inhibitors, whereas gatekeeper mutations modify the binding pocket to prevent drug binding.^2^,^8^,^9^ Off-target mechanisms include epithelial–mesenchymal transition, activation of alternative tyrosine kinase receptors, eg, EGFR, ERBB2/3, MET, and constitutional activation of alternative signalling pathways, ie, PI3K–Akt–mTOR, MAPK, JAK–STAT, and GSK3b.^2^,^10^,^11^

Futibatinib is an irreversible FGFR inhibitor that forms a covalent adduct to a conserved cysteine residue in the ATP pocket of FGFR, leading to improved potency and specificity.^4^ In vitro, futibatinib demonstrates activity against a wide range of mutations in the gatekeeper and molecular brake residues of the FGFR2 kinase domain.^10^ In the phase I trial of futibatinib in solid tumours, 17.9% (5/28) of patients with *FGFR2*-rearranged iCCA who had previously been treated with an FGFR inhibitor achieved a partial response, suggesting that this agent can overcome resistance mechanisms to earlier FGFR inhibitors.^8^

A case series of four patients with *FGFR2*-rearranged iCCA showed that treatment with futibatinib after progression on infigratinib or Debio1347 can lead to prolonged clinical benefit.^12^ A further case report of treatment with futibatinib after progression on pemigatinib described a partial response and progression-free survival of 23 months.^13^ Here, we present the case of a heavily pretreated patient with metastatic *FGFR2*–*PAWR* fusion–positive iCCA who achieved durable disease control with futibatinib after progression on a previous line of FGFR-targeting therapy.

## Case Presentation

**Figure 1:**
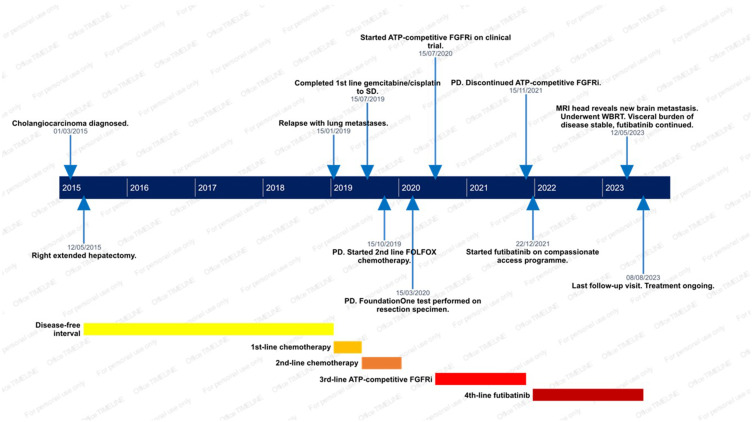
Timeline of events.

**Abbreviations**: SD, stable disease; PD, progression of disease; FOLFOX, folinic acid–5-fluorouracil–oxaliplatin; FGFRi, FGFR inhibitor; MRI, magnetic resonance imaging; WBRT, whole-brain radiotherapy.

A summary of the events is presented in Figure 1. A 50-year-old female with no pre-existing medical conditions presented in January 2015 with weight loss and bilirubinuria. CT imaging revealed a mass in the right liver lobe. Percutaneous liver biopsy confirmed a CK7^+^, CK20^–^ adenocarcinoma. She underwent a right extended hemihepatectomy with partial inferior vena cava excision. The final pathological staging was pT3 N0 (0/9 lymph nodes involved) R0 intrahepatic cholangiocarcinoma, with invasion of the inferior vena cava. No adjuvant chemotherapy was administered due to the lack of trial evidence supporting it at the time.

In January 2019, following a rise in her surveillance CA19-9, CT scanning detected distant disease relapse with three right lung metastases (1.5–5 cm in diameter). There was no locoregional relapse. CT-guided biopsy of the dominant right lung mass confirmed adenocarcinoma consistent with original biliary tract primary. She received 6 months of first-line chemotherapy with gemcitabine and cisplatin to stable disease. Disease progression with enlarging right lung metastases occurred 3 months after completion of first-line chemotherapy, and second-line chemotherapy with 5-fluorouracil–oxaliplatin–folinic acid was commenced. Restaging after 12 cycles (6 months) demonstrated progressive disease in March 2020.

At this stage, the patient remained asymptomatic from her disease, with an ECOG performance status of 0. As part of a clinical trial prescreening, the hepatic tumour resection specimen from 2015 was analysed using the FoundationOne CDx targeted next-generation sequencing assay.^14^ An *FGFR2*–*PAWR* fusion was identified on RNA-based fusion analysis. This is a rare *FGFR2* fusion accounting for <10% of *FGFR2*-rearranged iCCAs, and its effect on protein function is unknown.^11^ A nonsense co-mutation in the *BAP1* gene (p. Q40*) was identified on concurrently run DNA-based multitarget next-generation sequencing.

In June 2020, after a delay due to the COVID-19 pandemic, she was referred to our centre for consideration of a different clinical trial. She was screened and enrolled on a phase II clinical trial employing an ATP-competitive FGFR inhibitor for patients with chemotherapy-pretreated *FGFR2* fusion–positive biliary tract cancer. Baseline CT at this stage identified multiple right-sided lung metastases, the dominant being an irregular, necrotic 9.8×7.6 cm mass contacting the pulmonary vein and pericardium medially. Baseline CA19-9 was 173. The trial drug was commenced at a standard dose in July 2020.

She remained on this trial for 16 months, receiving 19 consecutive 4-weekly cycles of the investigational ATP-competitive FGFR inhibitor until November 2021. At that point, CT restaging showed enlarging right lung metastases, with the dominant right base mass extending via the right pulmonary vein into the right atrium. Anticoagulation with low–molecular weight heparin was commenced, as based on imaging only it was not possible to distinguish whether such CT appearances were due only to tumour thrombus or tumour infiltration plus superimposed benign thrombus. Disease progression was confirmed according to RECIST 1.1 criteria. CA19-9 had risen to 732. Treatment-related adverse events due to the investigational ATP-competitive FGFR inhibitor included grade 1 xerostomia/xerophthalmia, grade 1 hypercalcaemia, and a grade 1 ALT elevation. There were no treatment suspensions or dose reductions.

An application for the futibatinib expanded-access programme (NCT04507503) was accepted. Futibatinib was commenced at the standard dose of 20 mg once daily in December 2021. Following treatment initiation, she completed 24 3-weekly cycles (17 months on treatment) to May 2023. Consecutive 12-weekly response-evaluation CT scans each revealed minor improvement in disease burden and no new lesions up to that point.

**Figure 2:**
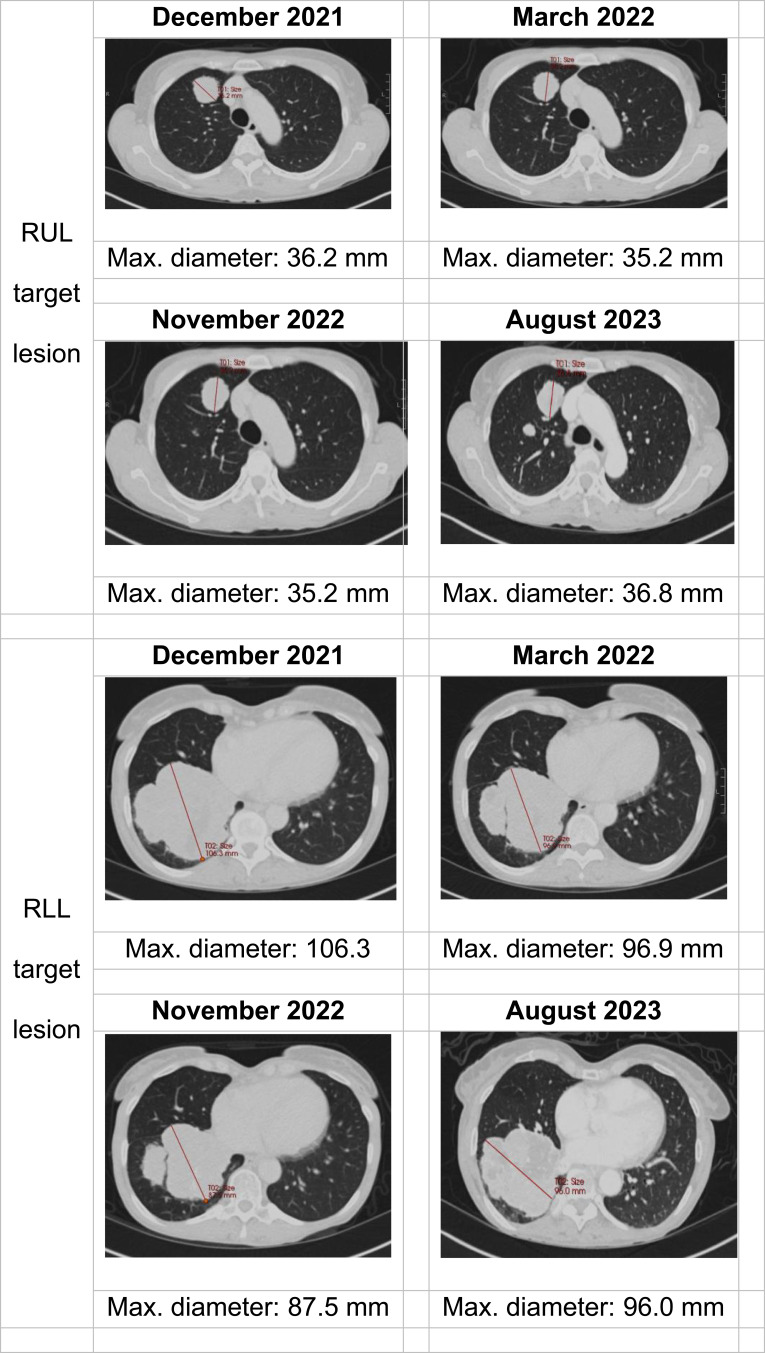
Serial computed tomography imaging of the chest. The images portray the target right upper lobe (RUL) and right lower lobe (RLL) lesions at baseline (progression on previous ATP-competitive FGFR inhibitor, prior to starting futibatinib) at the first response evaluation time point (3 months after commencing futibatinib), at the time of maximum tumour shrinkage (12 months after commencing futibatinib), and at the present time (20 months after commencing futibatinib).

**Abbreviations**: RUL, right upper lobe; RLL, right lower lobe.

Prior to commencing futibatinib, the dominant right base mass measured 11×10 cm (Figure 2). The maximum reduction in the sum of diameters in target lesions from baseline was 16% 12 months after treatment initiation (November 2022). CA19-9 nadir was 219 in August 2022. In May 2023, the patient developed new-onset confusion and was admitted to her local hospital. Neuroimaging demonstrated a large left-sided frontal lesion with associated vasogenic oedema. She was commenced on high-dose dexamethasone. Her symptoms rapidly improved on steroids, and she underwent whole-brain radiotherapy. Within 3 weeks, she had recovered to ECOG performance status 0 and was successfully weaned off steroids. Restaging CT of the chest, abdomen, and pelvis revealed largely stable intrathoracic disease burden and no new visceral metastases. Futibatinib is being continued (currently receiving cycle 29).

Treatment-related adverse events on futibatinib have included persistent grade 1–2 hypercalcaemia, grade 1 xerostomia, and intermittent grade 1 hyperphosphataemia. Adjusted calcium has been between 2.62 and 3.02 mmol/L between January 2022 and the present day. The patient has never developed symptoms from hypercalcaemia and has been managed with 3-weekly zoledronic acid infusions since April 2022. Serum phosphate has been found to have intermittent grade 1 elevations (maximum 1.68 mmol/L), but has never required intervention with phosphate binders. No treatment suspensions or dose reductions have been required for drug-related toxicities.

## Discussion

This case report adds to the literature supporting the possibility of durable tumour control with futibatinib after progression on ATP-competitive FGFR inhibitors.

Clinical benefit with futibatinib after the use of alternative ATP-competitive FGFR inhibitors has previously been noted in a case series of four patients,^12^ in a subgroup analysis of a phase I trial of futibatinib,^8^ and in a case report.^13^ These cases are summarised in Table 1. Our experience corroborates these findings and supports the use of futibatinib as a treatment strategy for patients with *FGFR2*-rearranged iCCA who have progressed on cytotoxic chemotherapy and an ATP-competitive FGFR inhibitor.

**Table 1:** Summary of previously reported cases

Study	Number of cases	Prior FGFR Inhibitor	PFS on prior FGFR inhibitor (months)	Best response on futibatinib	PFS range on futibatinib (months)
Goyal et al^12^	4	Infigratinib (n=3); Debio1347 (n=1)	5.6–12.6	2 SD, 2 PR	5.1–17.2
Meric-Bernstam et al^8^	5	Infigratinib (n=3); pemigatinib followed by infigratinib (n=1); PRN1371 (n=1).	NR	All PR	4.8–15.9
Rengan et al^13^	1	Pemigatinib	23	PR	23.6

**Abbreviations**: PFS, progression-free survival; NR, not reported; SD, stable disease; PR, partial response.

**Figure 3:**
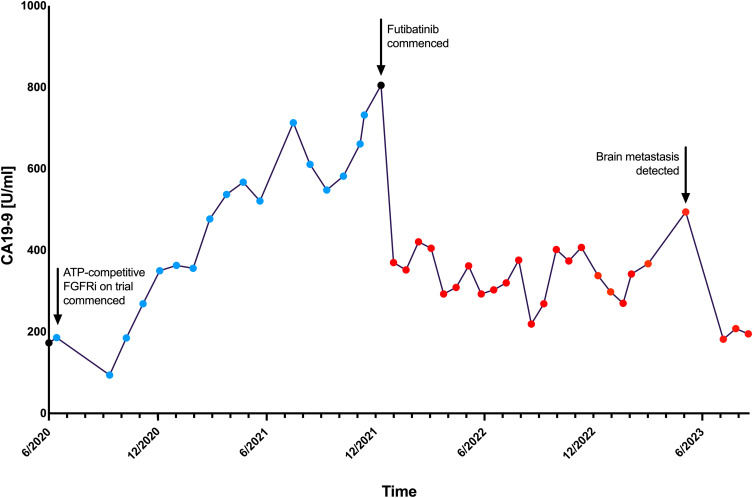
Graph representing serial CA19-9 levels during treatment with previous ATP-competitive FGFR inhibitor and with futibatinib.

**Abbreviation**: CA19-9, cancer antigen 19–9.

The duration of clinical benefit on both the prior FGFR inhibitor (16 months) and futibatinib (20 months) has been remarkable, considering that across trials of FGFR inhibitors in *FGFR2*-rearranged iCCA, time on treatment averages 7–9 months. Futibatinib elicited a marked biochemical response (CA19-9 concentration halved from approximately 800 to less than 400 over the first 3-weekly cycle), which had not been the case for the previous FGFR inhibitor (Figure 3).

Toxicities have been mild and easily manageable. Overall, futibatinib has been well tolerated and has allowed her to maintain her quality of life.

The patient experienced low-grade hypercalcaemia and hyperphosphataemia: these derive from inhibition of FGFR1 (most compounds in late clinical development have pan-FGFR-inhibitory activity; FGFR2-selective agents are in early drug development). The FGF23–FGFR1 signalling pathway inhibits sodium–phosphate co-transporters in the proximal renal tubule, thus limiting phosphate reabsorption. Additionally, FGF23–FGFR1 blocks the conversion of 25-hydroxyvitamin D to its active form, thereby limiting intestinal absorption of calcium and phosphate. FGFR inhibitors disrupt these processes, leading to hypercalcaemia and hyperphosphataemia.^15^ Trials of FGFR inhibitors have reported rates of hyperphosphataemia of 55%–85%.^5–9^ Phosphate-lowering therapies are recommended for moderate–severe cases (serum phosphate >1.78 mmol/l),^15^ but our patient did not require them as hyperphosphataemia never exceeded grade 1.

Should access to futibatinib not have been available, alternative options would have included consideration of locally available clinical trials, rechallenge with gemcitabine–cisplatin, which had induced disease control for 9 months in 2019, or irinotecan-based chemotherapy.^16^ Administering two consecutive lines of targeted therapy has allowed deferral of further cytotoxic therapy, which would have likely been associated with significant toxicity. No repeat tumour biopsy or circulating tumour DNA (ctDNA) analysis was performed upon progression on the previous line of FGFR-targeting therapy, as this was not required to access the futibatinib expanded-access programme (NCT04507503). Equally, the subsequent brain metastasis was treated with radiotherapy, and thus tissue biopsy at progression was not possible. However, repeat biopsies and sequencing on each progression may help to improve our understanding of the mechanisms responsible for the emergence of resistance to FGFR inhibitors.

Isolated central nervous system (CNS) progression on futibatinib has not previously been reported. The brain-to-plasma partition coefficient for futibatinib is unknown, and data for other FGFR inhibitors are sparse and indicate relatively modest CNS penetration.^17–20^ In oncogene-addicted non–small cell lung cancer, isolated CNS progression is associated with significantly lower detection of driver and resistance alterations in ctDNA as opposed to extra-CNS progression.^21^ Whether this finding is transferable to *FGFR2* fusion–positive cholangiocarcinoma remains unclear. When visceral progression becomes apparent, it will be useful to reperform next-generation sequencing on blood and/or tissue so as to clarify resistance mechanisms and guide future treatment in the light of a fast-evolving therapeutic landscape.

In oncogene-addicted non–small cell lung cancer, using newer-generation tyrosine kinases upfront, rather than administering them after progression on earlier-generation agents, leads to better overall survival.^22^,^23^ Newer FGFR inhibitors (RLY-4008, KIN-3248) with the potential to circumvent a variety of acquired resistance mechanisms are being investigated in phase I/II trials.^24–26^ A case of RLY-4008 causing significant tumour shrinkage after progression on futibatinib with development of an *FGFR2* p. E566V point mutation has been presented recently.^27^ However, the best sequence of these FGFR inhibitors in iCCA remains to be investigated in ongoing and future trials.

## Conclusion

FGFR inhibition has proven transformative for patients with *FGFR2* fusion–positive iCCA, who would otherwise be treated with standard cytotoxic chemotherapy and have median overall survival in the region of 12 months. This case portrayed how a subset of patients can derive prolonged clinical benefit from multiple lines of FGFR inhibitors. As FGFR inhibitors move forward toward the first-line therapy setting and the cholangiocarcinoma treatment arsenal incorporates chemoimmunotherapy, future trials in this space need to effectively address questions regarding the optimal sequencing of therapies including FGFR inhibitors and the combinations used.
